# Supplementary material for: PPM1D suppresses p53-dependent transactivation and cell death by inhibiting the Integrated Stress Response
Source: Nat Commun. 2022 Dec 1;13:7400. doi: 10.1038/s41467-022-35089-5 (PMC9715646; doi:10.1038/s41467-022-35089-5)
Supplement: Supplementary file 1 — Supplementary Information [file 41467_2022_35089_MOESM1_ESM.pdf]

Supplementary Figures.

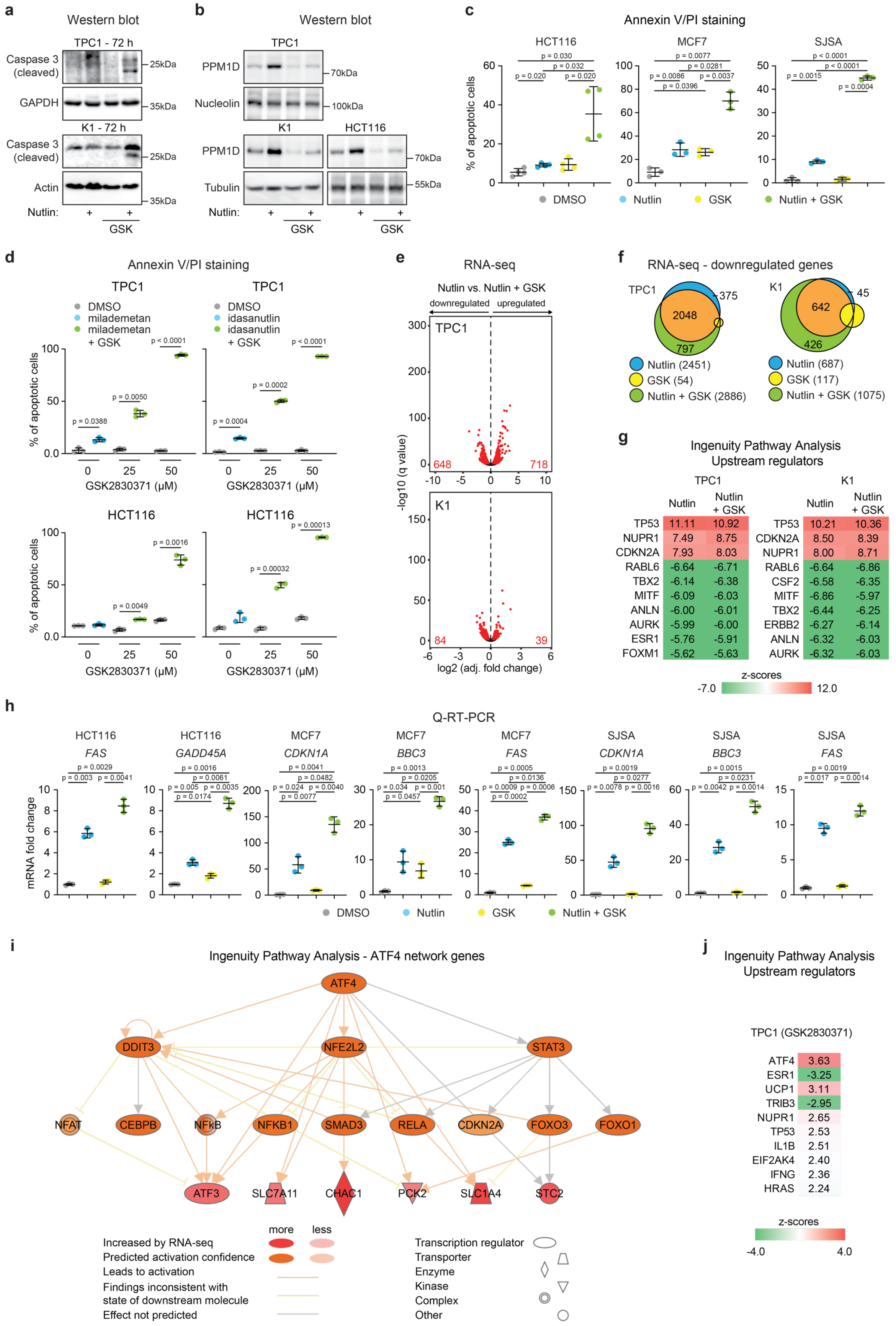

Supplementary Fig. 1. Combined inhibition of MDM2 and PPM1D leads to increased expression of p53 target genes and apoptosis, related to Fig. 1.

a, b Western blots of TPC1, K1, and HCT116 cells treated with vehicle (0.2% dimethyl sulfoxide, DMSO), 10 μM nutlin-3a, 25 μM GSK2830371 (GSK), or the drug combination for 24 hours. Results shown in **a** and **b** are representative of three independent experiments. **c** HCT116 (n = 4 independent experiments), MCF7 (n = 3 independent experiments), and SJSA (n = 3 independent experiments) cells were treated as in **b** for 72 hours. Harvested cells were stained with Annexin V-FITC/PI and analyzed by flow cytometry. Data are represented as mean ± SD. Paired, two-sided t test was used to calculate the indicated p value, n = 3 independent experiments. **d** TPC1 and HCT116 cells were treated with indicated compounds for 48 hours. After the treatment cells were harvested by trypsinization, stained with Annexin V-FITC/PI, and analyzed by flow cytometry. Data are represented as mean ± SD. Paired, two-sided t test was used to calculate the indicated p value, n = 3 independent experiments. **e** Volcano plots of differentially expressed genes identified by RNA-seq. Red data points and numbers indicate significantly up- and downregulated genes ($q < 0.05$, adjusted fold change > 1.5) in cells treated with the drug combination when compared to nutlin alone. **f** Overlaps among indicated groups of downregulated genes. **g** Prediction of upstream regulators by Ingenuity Pathway Analysis in genes upregulated by nutlin and the drug combination ($q < 0.05$, adjusted fold change > 1.5). **h** Q-RT-PCR analysis of p53 target genes in HCT116, MCF7, and SJSA cell lines treated for 24 hours with indicated compounds. Data are represented as mean ± SD. Paired, two-sided t test was used to calculate the indicated p value, n = 3 independent experiments. **i** ATF4 network and regulatory links as depicted by Ingenuity Pathway Analysis. Positions of genes with FC > 5 for Nutlin + GSK2830371 vs. Nutlin comparison in the network are shown. **j** Upstream regulators predicted by Ingenuity Pathway Analysis in genes upregulated by GSK2830371 only in TPC1 cells ($q < 0.05$, adjusted fold change > 1.5). Source data are provided as a Source Data file.

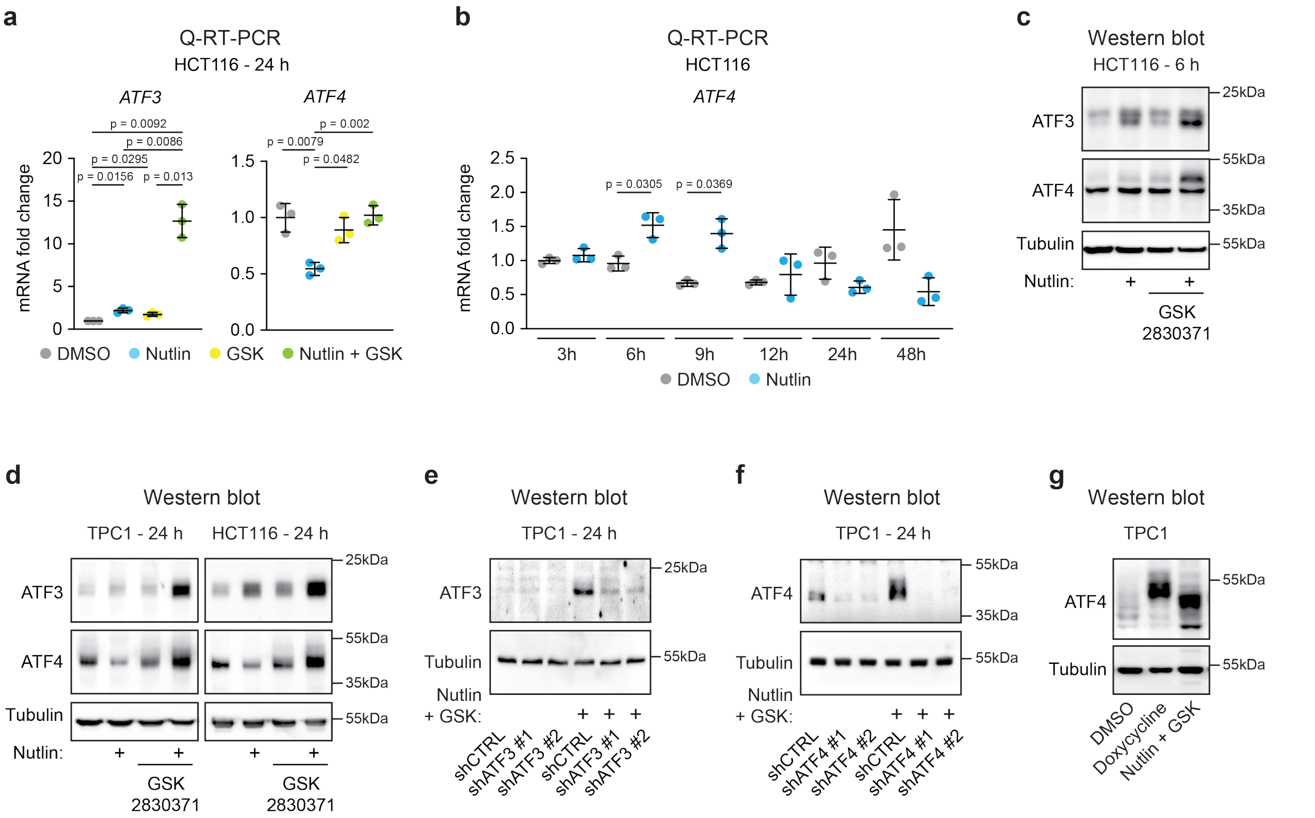

Supplementary Fig. 2. ATF4 is induced by dual inhibition of MDM2 and PPM1D, related to Fig. 2.

a Q-RT-PCR of *ATF3* and *ATF4* mRNA in HCT116 cells lines treated with vehicle (0.2% DMSO), 10 μM nutlin-3a, 25 μM GSK2830371 (GSK), or the drug combination for 24 hours (n = 3 independent experiments). **b** Q-RT-PCR analysis of *ATF4* mRNA in TPC1 cells treated with 10 μM nutlin-3a for indicated time points (n = 3 independent experiments). **c-g** Western blots of cells exposed to indicated compounds for either 6 (**c**) or 24 hours (**d-g**). In **e-f**, cells were transduced with non-targeting control shRNAs (shCTRL), or shRNAs targeting ATF3 or ATF4. In **g**, doxycycline was used at 10 μg/ml for 24 hours to induce ATF4 expression. Data in a and b are represented as mean ± SD, n = 3 independent experiments. Paired, two-sided t test was used to calculate the indicated p values. Source data are provided as a Source Data file.

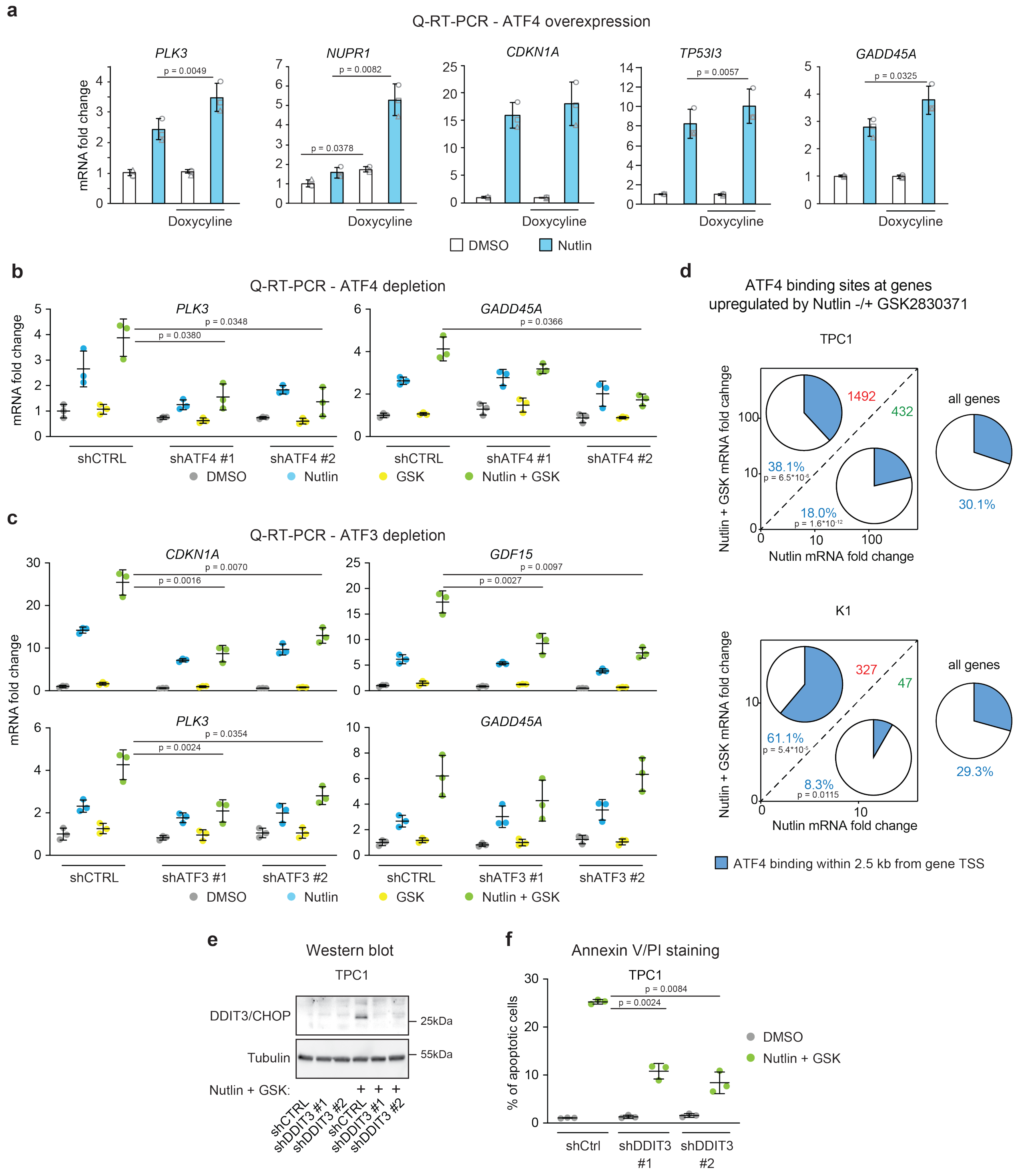

Supplementary Fig. 3. ATF4 and ATF3 are required for increased induction of p53 target genes, related to Fig. 2. a-c Q-RT-PCR analysis of p53 target genes in TPC1 cells expressing recombinant ATF4 from a Tet-on vector upon induction with 10 μg/ml doxycycline for 24 hours (**a**), depleted of ATF4 (**b**), or depleted of ATF3 (**c**) (n = 3 independent experiments in **a**, **b**, and **c**). Paired, two-sided t test was

used to calculate the indicated p value. Data are represented as mean ± SD. **d** Analysis of ATF4 binding sites frequency at transcription start sites (TSS) of genes detected by RNA-seq in TPC1 and K1 cell lines. ATF4 binding loci in cell line K562 were obtained from ENCODE ChIP-seq dataset ENCSR044UJJ. Indicated p vales were calculated with hypergeometric test in binding frequency when compared to ATF4 ChIP-seq read accumulation across all detected genes in the respective cell line. The quadrant above the dashed line indicates genes over-induced upon combinatorial treatment with nutlin-3a and GSK283037 (GSK). **e** Western blot of cells transduced with non-targeting control shRNAs (shCTRL), or shRNAs targeting DDIT3/CHOP. **f** TPC1 cells were treated with vehicle (0.2% DMSO) or combination of 10 μM nutlin-3a and 25 μM GSK283037 for 72 hours. Harvested cells were stained with Annexin V-FITC/PI and analyzed by flow cytometry. Data are represented as mean ± SD. Paired**,** two-sided t test was used to calculate the indicated p value, n = 3 independent experiments. Source data are provided as a Source Data file.

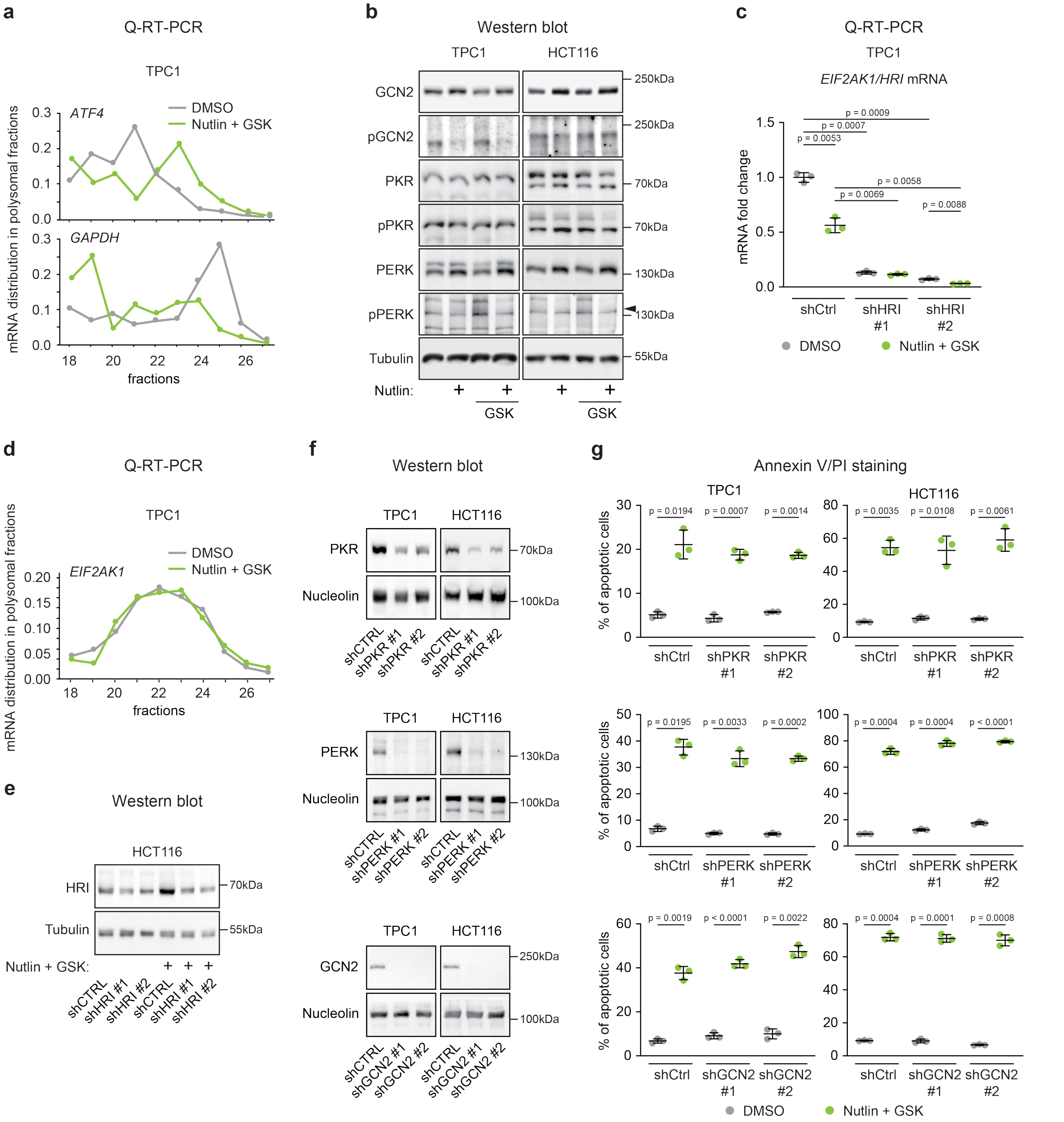

Supplementary Fig. 4. Analysis of signaling upstream of eIF2α phosphorylation, related to Fig. 3. **a** Q-RT-PCR of *ATF4* and *GAPDH* mRNAs in RNA samples isolated from polysome fractions presented in Fig. 3c. TPC1 cells were treated with vehicle (0.2% dimethyl sulfoxide, DMSO) or combination of 10 μM nutlin-3a and 25 μM GSK283037 (GSK) for 24 hours. mRNA distribution profiles show representative results of three independent experiments. **b** Western blots of TPC1 and HCT116 cells treated with vehicle

(0.2% DMSO), 10 μM nutlin-3a, 25 μM GSK2830371, or the drug combinations for 24 hours. **c** Q-RT-PCR of *EIF2AK1* (HRI) in TPC1 cells expressing shRNAs targeting *EIF2AK1* treated for 24 hours with tested compounds as indicated. Paired**,** two-sided t test was used to calculate the indicated p value., n = 3 independent experiments. **d** Q-RT-PCR of *EIF2AK1* mRNA in RNA samples isolated from polysome fractions as in **a**. *EIF2AK1* mRNA distribution profile shows a representative result of three independent experiments. **e** Western blots of HCT116 cells depleted of HRI and treated with indicated compounds for 24 hours. **f** Western blots of TPC1 and HCT116 cells depleted of PKR, PERK, and GCN2 kinases with two different shRNAs. **g** TPC1 and HCT116 cells expressing the indicated shRNAs were treated with vehicle (0.2% DMSO) or combination of 10 μM nutlin-3a and 25 μM GSK283037 for 72 hours. Harvested cells were stained with Annexin V-FITC/PI and analyzed by flow cytometry. Data in **c** and **g** are represented as mean ± SD, n = 3 independent experiments. Paired**,** two-sided t test was used to calculate the indicated p value. Source data are provided as a Source Data file.

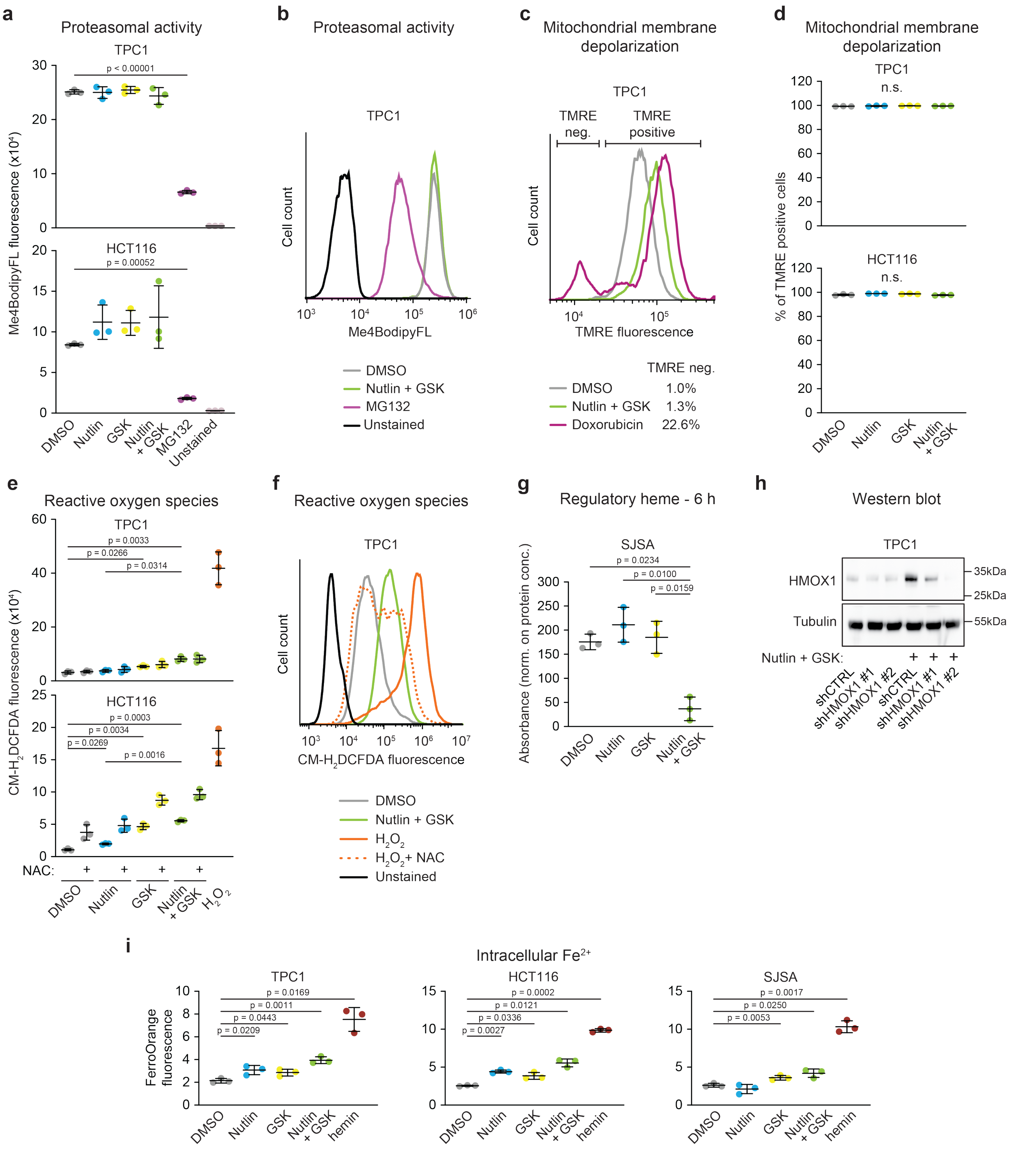

Supplementary Fig. 5. Analysis of signaling upstream of eIF2α phosphorylation, related to Fig. 3.

a, b Flow cytometric analysis of TPC1 and HCT116 cells treated for 6 hours with vehicle (0.2% DMSO), 10 μM nutlin-3a, 25 μM GSK2830371 (GSK), or the drug combination and stained using Me4BodipyFL-Ahx3Leu3VS proteasome activity probe. 6-hour treatment with 1 μM MG132 was used as a positive

control. **c, d** Mitochondrial membrane potential ($\Delta\Psi$m) analysis using the fluorescent probe TMRE and flow cytometry. TPC1 and HCT116 cells were treated with vehicle (0.2% DMSO), 10 μM nutlin-3a, 25 μM GSK2830371, or the drug combinations for 24 hours. 1 μM doxorubicin (48 hours) was used as a positive control in **c** to document fluorescence of a cell population with depolarized mitochondrial membrane [tetramethylrhodamine, ethyl ester (TMRE) negative]. Fraction of TMRE positive cells (no mitochondria depolarization) was plotted in **d**. n.s. indicates no significant differences for any of the treatment relative to DMSO (paired, two-sided t test, n = 3 independent experiments). **e, f** Reactive oxygen species measurement by flow cytometry. Cells treated with indicated compounds for 6 hours were harvested and stained with the chloromethyl 2',7'-dichlorodihydrofluorescein diacetate (CM-H_2DCFDA) probe. 2.5 mM N-acetylcysteine (NAC) was used with tested compounds where indicated. Hydrogen peroxide served as a positive control (10 mM, 30 minutes). **g** Cellular levels of free (regulatory) heme were measured in SJSA cells treated with denoted compounds for 6 hours. **h** Western blot. TPC1 cells depleted of HMOX1 were treated as in **a**. **i** Intracellular levels of Fe^{2+} were measured by flow cytometry using fluorescent probe FerroOrange. Cells were treated as in **d** and 50 μM hemin was used as a positive control. Paired, two-sided t test was used in **a**, **e**, **g**, and **i** to calculate the indicated p value. Data in **a**, **d**, **e**, **g**, and **i** are represented as mean ± SD, n = 3 independent experiments. Source data are provided as a Source Data file.

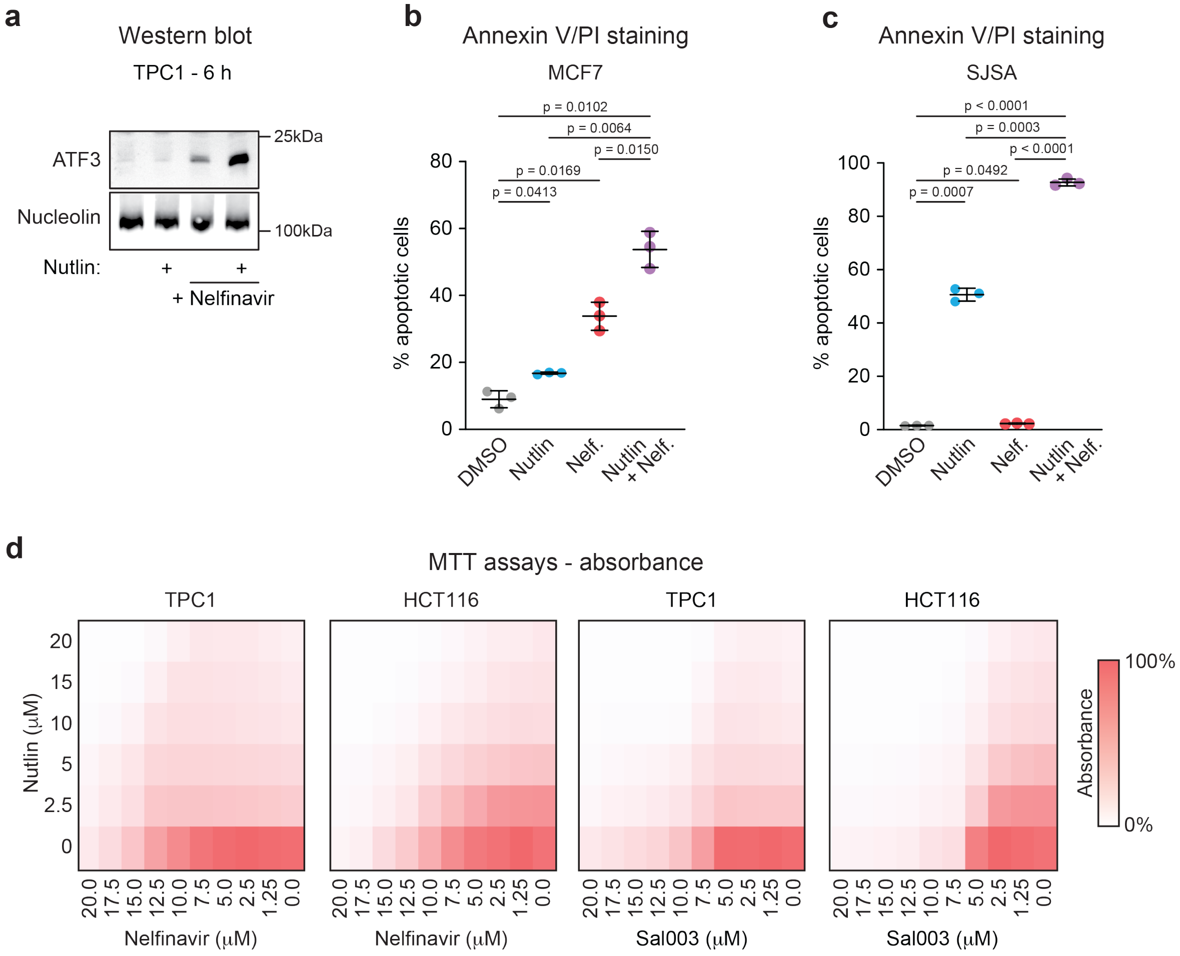

Supplementary Fig. 6. Synergistic effects of nutlin-3a and pharmacological inhibitors of eIF2α, related to Fig. 4. a TPC1 cells were treated with vehicle (0.2% DMSO), 10 μM nutlin-3a, 20 μM nelfinavir, or the drug combination for 6 hours. **b, c** MCF7 and SJSA cells were treated with compounds and concentrations indicated in (**a**) for 48 hours. After the treatment period, cells were harvested, stained with Annexin V-FITC/PI, and analyzed by flow cytometry. Data are represented as mean ± SD (n = 3 independent experiments). Statistical significance was calculated by paired**,** two-sided t test. **d** Averages (n = 3 independent experiments) of MTT test absorbances measured at 570 nm plotted as heatmaps. TPC1 and HCT116 cells were treated with indicated compounds for 72 hours. Source data are provided as a Source Data file.

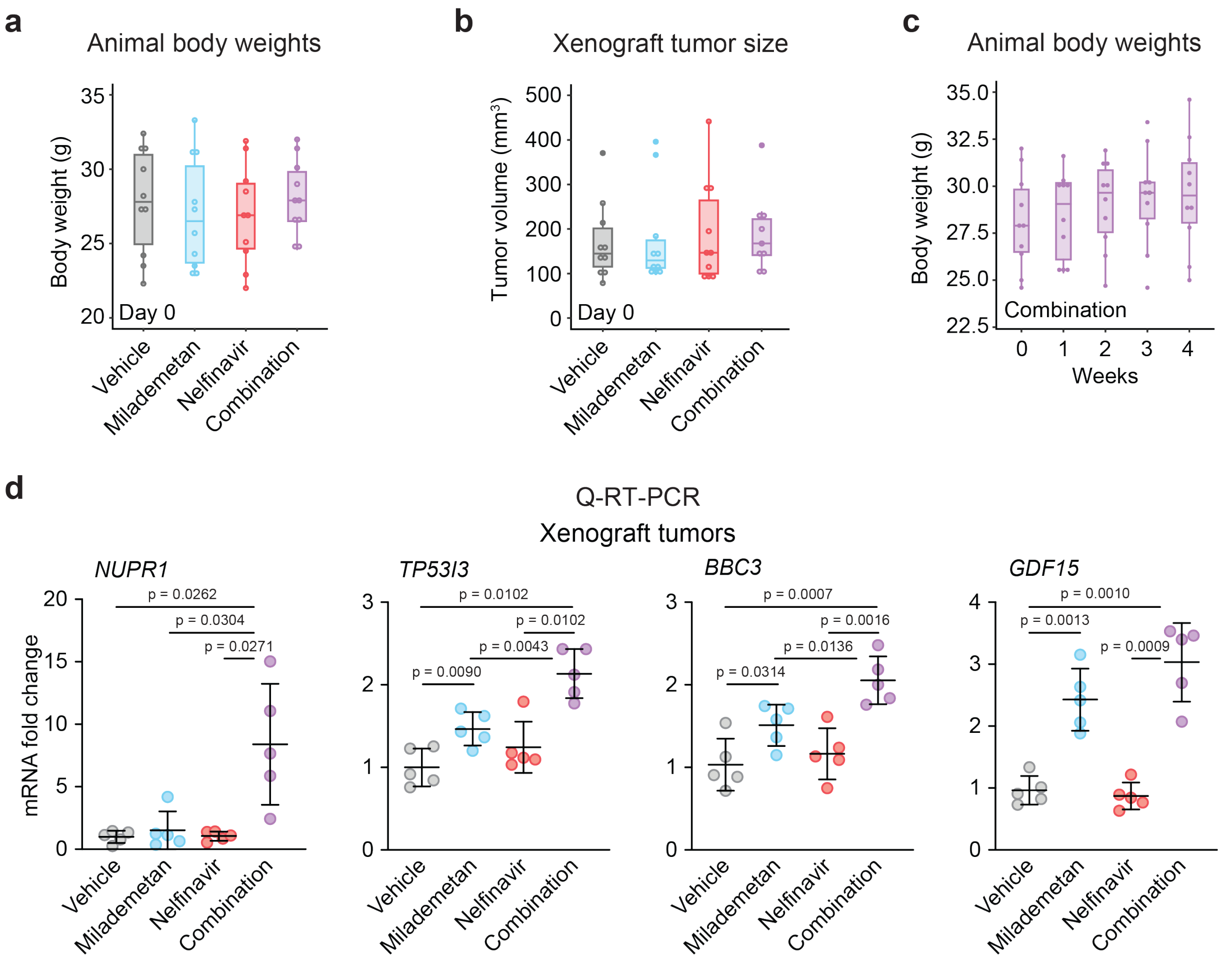

Supplementary Fig. 7. HCT116 tumor xenograft, related to Fig. 5. a Body weights of experimental animals at the time of treatment initiation (n = 10 animals per group). **b** Initial tumor sizes across treatment groups (n = 10 animals per group). **c** Body weights of experimental animals from the group treated with the drug combination (n = 10 animals per group). Data points represent initial body weights (week 0) and values at days 7, 14, 21, and 28, respectively. Box plots center lines in **a**, **b**, and **c** represent median values, box boundaries outline the 25th and 75th percentile. Whiskers depict the smallest or largest values within 1.5 times of the interquartile range. **d** Relative mRNA levels of p53 target genes analyzed by Q-RT-PCR using RNA extracted from tumors. Data are represented as mean ± SD. Unpaired**,** two-sided t test was used for calculations of statistical significance (n = 5 tumor samples from 5 individual animals per group). Source data are provided as a Source Data file.

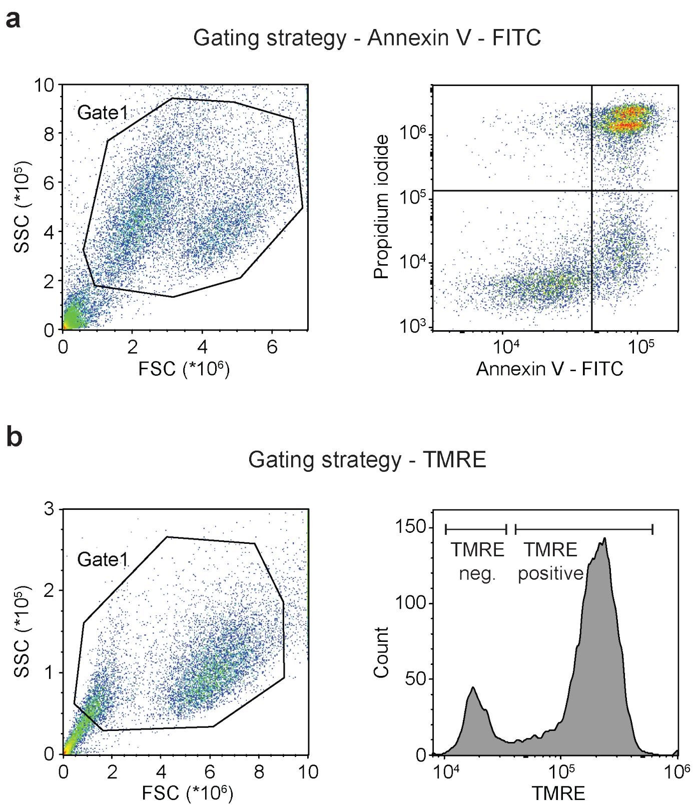

Supplementary Fig. 8. Flow cytometry gating strategy. Cell-sized particles in Gate 1 were selected for further analysis of populations stained with annexin V-FITC/propidium iodide (**a**) and TMRE (**b**). Cell suspensions stained with CM-H_2DCFDA, Me4BodipyFL, and FerroOrange were gated for cell-sized particles as in **a**.
