# Supplementary material for: PPM1D suppresses p53-dependent transactivation and cell death by inhibiting the Integrated Stress Response
Source: Nat Commun. 2022 Dec 1;13:7400. doi: 10.1038/s41467-022-35089-5 (PMC9715646; doi:10.1038/s41467-022-35089-5)
Supplement: Supplementary file 3 — Description of additional Supplementary File [file 41467_2022_35089_MOESM3_ESM.pdf]

Descriptions of Additional Supplementary Data Files

Supplementary Data 1. RNAseq results. Results of DESeq2 analysis of transcriptome data of two different cell lines (TPC1 and K1) treated with DMSO (vehicle), Nutlin, GSK2830371, and combination of both drugs.

Supplementary Data 2. Ingenuity Pathway Analysis of RNA-seq data. Upstream Regulator Prediction in TPC1 and K1 cell lines treated with DMSO (vehicle), Nutlin, GSK2830371, and combination of both drugs. Upstream regulators with z-score >1.3 and <-1.3 are displayed.

Supplementary Data 3. Oligonucleotides and antibodies. Lists of oligonucleotides used for qRTPCR, ATF4 cloning, ChIP-qPCR, and lists of antibodies used in this study.
