# Supplementary material for: PPM1D suppresses p53-dependent transactivation and cell death by inhibiting the Integrated Stress Response
Source: Nat Commun. 2022 Dec 1;13:7400. doi: 10.1038/s41467-022-35089-5 (PMC9715646; doi:10.1038/s41467-022-35089-5)
Supplement: Supplementary file 7 — Reporting Summary [file 41467_2022_35089_MOESM7_ESM.pdf]

nature portfolio

Corresponding author(s): Joaquin M Espinosa

Last updated by author(s): Nov 13, 2022

Reporting Summary

Nature Portfolio wishes to improve the reproducibility of the work that we publish. This form provides structure for consistency and transparency in reporting. For further information on Nature Portfolio policies, see our Editorial Policies and the Editorial Policy Checklist.

Statistics

For all statistical analyses, confirm that the following items are present in the figure legend, table legend, main text, or Methods section.

n/a	Confirmed	
☐	☒	The exact sample size (*n*) for each experimental group/condition, given as a discrete number and unit of measurement
☐	☒	A statement on whether measurements were taken from distinct samples or whether the same sample was measured repeatedly
☐	☒	The statistical test(s) used AND whether they are one- or two-sided<br>*Only common tests should be described solely by name; describe more complex techniques in the Methods section.*
☒	☐	A description of all covariates tested
☐	☒	A description of any assumptions or corrections, such as tests of normality and adjustment for multiple comparisons
☐	☒	A full description of the statistical parameters including central tendency (e.g. means) or other basic estimates (e.g. regression coefficient) AND variation (e.g. standard deviation) or associated estimates of uncertainty (e.g. confidence intervals)
☐	☒	For null hypothesis testing, the test statistic (e.g. *F*, *t*, *r*) with confidence intervals, effect sizes, degrees of freedom and *P* value noted<br>*Give P values as exact values whenever suitable.*
☒	☐	For Bayesian analysis, information on the choice of priors and Markov chain Monte Carlo settings
☒	☐	For hierarchical and complex designs, identification of the appropriate level for tests and full reporting of outcomes
☒	☐	Estimates of effect sizes (e.g. Cohen's *d*, Pearson's *r*), indicating how they were calculated

Our web collection on statistics for biologists contains articles on many of the points above.

Software and code

Policy information about availability of computer code

Data collection	Flow cytometry: Accuri c6 (version 1.0.264.21)<br>Immunohistochemistry: InForm Vectra (version 3.0.5)<br>MTT assays: SynergyFinder (version 2.0)
Data analysis	Flow cytometry: Accuri c6 (version 1.0.264.21), FlowJo (visualization, version 10.4.2)<br>Immunohistochemistry: InForm (version 2.6)<br>RNA-seq data processing and analysis: FASTQC (version 0.11.2), FastQ Screen (v0.4.4), Fastx toolkit (v0.0.13.2), TopHat2 (v2.0.13), SAMtools (v0.1.19), Picard (v1.129), RSeQC (v2.6), HTSeq (v0.6.1), DESeq2 (version 1.6.3), R (version 3.1.0), IGV genome viewer (v2.8.10)<br>ChIP-seq data processing and analysis: FASTQC (v0.11.5), FastQ Screen (v0.11.0), FASTQ-MCF from EAUtils (v1.05), Bowtie2 (v2.2.9), Samtools (v1.5), Picard (v2.9.4), deepTools105 (version 2.2.2)

For manuscripts utilizing custom algorithms or software that are central to the research but not yet described in published literature, software must be made available to editors and reviewers. We strongly encourage code deposition in a community repository (e.g. GitHub). See the Nature Portfolio guidelines for submitting code & software for further information.

Data

Policy information about availability of data

All manuscripts must include a data availability statement. This statement should provide the following information, where applicable:

- Accession codes, unique identifiers, or web links for publicly available datasets
- A description of any restrictions on data availability
- For clinical datasets or third party data, please ensure that the statement adheres to our policy

RNA-seq and ChIP-seq data generated in this study have been deposited to the Gene Expression Omnibus (GEO) database and are available under the accession number GSE191150 [https://www.ncbi.nlm.nih.gov/geo/query/acc.cgi?acc=GSE191150]. All other Source Data are provided with this paper in the Source Data files. This paper analyzed data from the Genotype-Expression Project (GTEx) [https://gtexportal.org/home/datasets], The Cancer Genome Atlas Project (TCGA) [https://www.cancer.gov/about-nci/organization/ccg/research/structural-genomics/tcga/using-tcga], as well as publicly available data for ATF4 chromatin binding [https://www.encodeproject.org/experiments/ENCSR044UJJ/], global run on-deep sequencing (GRO-seq) data and matching RNAseq data under conditions of p53 stimulation [https://www.ncbi.nlm.nih.gov/geo/query/acc.cgi?acc=GSE86222]. Microscopy images are shared at FigShare portal [https://figshare.com/articles/media/mouse_tumors_and_organoids/21545292]. Any additional information required to re-analyze data reported in this paper will be provided by the corresponding authors upon request.

Field-specific reporting

Please select the one below that is the best fit for your research. If you are not sure, read the appropriate sections before making your selection.

☒ Life sciences ☐ Behavioural & social sciences ☐ Ecological, evolutionary & environmental sciences

For a reference copy of the document with all sections, see nature.com/documents/nr-reporting-summary-flat.pdf

Life sciences study design

All studies must disclose on these points even when the disclosure is negative.

Sample size	Immunohistochemistry image analysis libraries were downsampled based on power analysis calculation using formula n = (Zsigma/E)2 where n is the sample size required to ensure that the margin of error (E, 95%) does not exceed the value specified as 25% of the vehicle-treated nuclei signal, Z is the value from the table of probabilities of the standard normal distribution for the desired confidence level, and sigma is the standard deviation of the outcome of interest. Other experiments used sample sizes (number of replicates) based on our previous studies using the same methods and experimental layouts (PMID: 22246181, PMID: 28904012, PMID: 23684607)
Data exclusions	In Q-RT-PCR experiments individual technical replicate values were excluded if meeting the criteria for an outlier (>50% difference from the average value of remaining replicates). This applied to ~2% of values.
Replication	All data can be reproduced. Any experiments which failed to reproduce were not used in this manuscript. Replicates were prepared independently (biological replicates) and technical replicate values were averaged and treated as one single measurement for statistical analysis. Sufficient cell numbers for flow cytometry were acquired (typically 10,000 per sample). All experiments were repeated (performed independently as biological replicates) at least three times.
Randomization	Mice bearing tumors were assigned to treatment groups randomly. No difference among the initial tumor size or animal body weights distributions were controlled for as reported in Supplementary Fig. 5a-b. All other experiments were based on tissue cultures where individual plates were assigned to treatment groups at random.
Blinding	Blinding of the xenograft experiment was not possible because of distinct drug solution appearance, and measurements performed at the time of treatment. In other experiments blinding does not apply since measurement were performed by instruments in one setting for all treatments/groups per experiment.

Reporting for specific materials, systems and methods

We require information from authors about some types of materials, experimental systems and methods used in many studies. Here, indicate whether each material, system or method listed is relevant to your study. If you are not sure if a list item applies to your research, read the appropriate section before selecting a response.

Materials & experimental systems

n/a	Involved in the study
	x Antibodies
	x Eukaryotic cell lines
x	Palaeontology and archaeology
	x Animals and other organisms
x	Human research participants
x	Clinical data
x	Dual use research of concern

Methods

n/a	Involved in the study
	x ChIP-seq
	x Flow cytometry
x	MRI-based neuroimaging

Antibodies

Antibodies used

Target; Clone; Source; Catalog number; Identifier
p53; DO-1; Calbiochem; OP43; AB_10682938
phospho-Ser15-p53; polyclonal; Cell Signaling Technology; #9284; AB_331464
beta-actin; C4; Santa Cruz Biotechnology; sc-47778; AB_2714189
alpha-tubulin; DM1A; Millipore Sigma; T9026; AB_477593
Nucleolin (C23); H6; Santa Cruz Biotechnology; sc-55486; AB_670272
GAPDH; G9; Santa Cruz Biotechnology; sc-365062; AB_10847862
PPM1D (WIP1); F10; Santa Cruz Biotechnology; sc-376257; AB_10986000
ATF3; A8; Santa Cruz Biotechnology; sc-518032; n/a
ATF4; B3; Santa Cruz Biotechnology; sc-390063; AB_2810998
p-eIF2a (p-Ser51); D9G8; Cell Signaling Technology; #3398; AB_2096481
Casp-3 (cleaved); polyclonal; Cell Signaling Technology; #9661; AB_2341188
HRI; 7H3L3; Invitrogen; 702551; AB_2664566
GCN2; F7; Santa Cruz Biotechnology; sc-374609; AB_10986130
p-GCN2 (p-Thr899); polyclonal; Novus; AF7605; AB_2843969
PKR; H12; Santa Cruz Biotechnology; sc-514626; n/a
p-PKR (p-Thr446); SY230; Novus; NBP2-67426; n/a
PERK; B5; Santa Cruz Biotechnology; sc-377400; AB_2762850
p-PERK (p-Thr980); G.305.4; Invitrogen; MA5-15033; AB_10980432
HO-1; 23; Santa Cruz Biotechnology; sc-136256; AB_2011615
Ki-67 (SP6); SP6; Epredia; RM-9106; AB_2341197
DDIT3/CHOP; L63F7; Cell Signaling Technology; #2895; AB_2089254
Puma; G3; Santa Cruz Biotechnology; sc-374223; AB_10987708
"Anti mouse IgG; HRP linked Antibody "; polyclonal; Cell Signaling Technology; #7076; AB_330924
"Anti rabbit IgG; HRP linked Antibody "; polyclonal; Cell Signaling Technology; #7074; AB_2099233
ATF4 (CHIP); B3; Santa Cruz Biotechnology; sc-390063X; AB_2810998
IgG (ChIP); polyclonal; Santa Cruz Biotechnology; sc-2025; AB_737182

Validation

Target; Reference(s); Validation by manufacturer; Validated for human protein; Validated for western blot; Validated for (chromatin) immunoprecipitation; Additional validations
p53; PMID: 7685617; "https://www.emdmillipore.com/US/en/product/Anti-p53-Ab-6-Pantropic-Mouse-mAb-DO-1; EMD_BIO-OP43"; Yes; Yes; n/a; by knock-out (PMID: 22246181)
phospho-Ser15-p53; "PMID:23913444; PMID:24064360; PMID:25560828"; https://www.cellsignal.com/products/primary-antibodies/phospho-p53-ser15-antibody/9284; Yes; Yes; n/a;
beta-actin; "PMID:28017329; PMID:29107503; PMID:29149593"; https://www.scbt.com/p/beta-actin-antibody-c4; Yes; Yes; n/a;
alpha-tubulin; "PMID:19425080; PMID:19757494; PMID:24684304"; https://www.sigmaaldrich.com/US/en/product/sigma/t9026; Yes; Yes; n/a;
Nucleolin (C23); PMID:28833137; https://www.scbt.com/p/c23-antibody-h-6; Yes; Yes; n/a;
GAPDH; "PMID:24971611; PMID:25892301; PMID:27504807"; https://www.scbt.com/p/gapdh-antibody-g-9; Yes; Yes; n/a;
PPM1D (WIP1); PMID:30388424; https://www.scbt.com/p/wip1-antibody-f-10; Yes; Yes; n/a; by knock-out (author's unpublished data)
ATF3; PMID:34855279 ; https://www.scbt.com/p/atf-3-antibody-a-8; Yes; Yes; n/a; by knock-down (this manuscript)
ATF4; PMID:36251357; https://www.scbt.com/p/atf-4-antibody-b-3; Yes; Yes; n/a; by knock-down (this manuscript)
p-eIF2a (p-Ser51); "PMID:26327577; PMID:27912058; PMID:27984728"; https://www.cellsignal.com/products/primary-antibodies/phospho-eif2a-ser51-d9g8-xp-rabbit-mab/3398; Yes; Yes; n/a;
Casp-3 (cleaved); "PMID:16736467; PMID:17099894; PMID:17299760"; https://www.cellsignal.com/products/primary-antibodies/cleaved-caspase-3-asp175-antibody/9661; Yes; Yes; n/a;
HRI; PMID:30894532; https://www.thermofisher.com/antibody/product/HRI-Antibody-clone-7H3L3-Recombinant-Monoclonal/702551; Yes; Yes; n/a; by knock-down (this manuscript)
GCN2; "PMID:36302756; PMID:33899121; PMID:33760196"; https://www.scbt.com/p/gcn2-antibody-f-7; Yes; Yes; n/a; by knock-down (this manuscript)
p-GCN2 (p-Thr899); ; https://www.novusbio.com/products/gcn2-antibody_af7605; Yes; Yes; n/a;
PKR; ; https://www.scbt.com/p/pkr-antibody-h-12; Yes; Yes; n/a; by knock-down (this manuscript)

p-PKR (p-Thr446); ; https://www.novusbio.com/products/pkr-antibody-sy230_nbp2-67426; Yes; Yes; n/a;
PERK; PMID:30937420; https://www.scbt.com/p/perk-antibody-b-5; Yes; Yes; n/a; by knock-down (this manuscript)
p-PERK (p-Thr980); "PMID:26219498; PMID:26927933; PMID:31081119"; https://www.thermofisher.com/antibody/product/Phospho-PERK-Thr980-Antibody-clone-G-305-4-Monoclonal/MA5-15033; Yes; Yes; n/a;
HO-1; PMID:29067120; https://www.scbt.com/p/heme-oxygenase-1-antibody-23; Yes; Yes; n/a;
Ki-67 (SP6); "PMID:28457749; PMID:28609656; PMID:28695822"; https://www.fishersci.com/shop/products/ki-67-rabbit-monoclonal-antibody/RM9106S1; Yes; n/a; n/a;
DDIT3/CHOP; "PMID:24265448; PMID:28938442; PMID:29107503"; https://www.cellsignal.com/products/primary-antibodies/chop-l63f7-mouse-mab/2895; Yes; Yes; n/a; by knock-down (this manuscript)
Puma; "PMID:30561326; PMID:30712844; PMID:30850922"; https://www.scbt.com/p/pumaalpha-beta-antibody-g-3; Yes; Yes; n/a;
"Anti mouse IgG; HRP linked Antibody "; "PMID:24828612; PMID:25546454; PMID:26335297"; https://www.cellsignal.com/products/secondary-antibodies/anti-mouse-igg-hrp-linked-antibody/7076; n/a; Yes; n/a;
"Anti rabbit IgG; HRP linked Antibody "; "PMID:23970788; PMID:24140712; PMID:24828612"; https://www.cellsignal.com/products/secondary-antibodies/anti-mouse-igg-hrp-linked-antibody/7074; n/a; Yes; n/a;
ATF4 (CHIP); PMID: 30452882; https://www.scbt.com/p/atf-4-antibody-b-3; Yes; n/a; Yes; by knock-down (this manuscript)
IgG (ChIP); "PMID:25663431; PMID:26996949; PMID:28123016"; https://www.scbt.com/p/normal-mouse-igg; n/a; n/a; Yes;
ENCODE PROJECT External validation for lot# C111 is available under ENCODE ID: ENCAB615SBV

Eukaryotic cell lines

Policy information about cell lines

Cell line source(s)	TPC1, Dr. Rebecca Schweppe - Gift; Sigma-Aldrich SKU:SCC147, RRID: CVCL_6298<br>K1, Dr. Rebecca Schweppe, Gift; Sigma-Aldrich SKU: 92030501-1VL, RRID: CVCL_2537<br>HCT116, Dr. Bert Vogelstein - Gift; Sigma Aldrich SKU:9109005-1VL, RRID: CVCL_0291<br>HEK293FT, Dr. Beverly Emerson - Gift; Thermo Fisher, cat. # R70007, RRID: CVCL_6911<br>MCF7, Dr. Beverly Emerson - Gift; Sigma-Aldrich SKU: SCC100, RRID: CVCL_0031<br>SJSA1, Dr. Beverly Emerson - Gift; ATCC Catalog Number CRL-2098, RRID: CVCL_1697
Authentication	STR authentication
Mycoplasma contamination	PCR test, only cultures with negative results were used in the study
Commonly misidentified lines (See ICLAC register)	K1 cell line is a GLAG-66 derivative as acknowledged in Methods section.

Animals and other organisms

Policy information about studies involving animals; ARRIVE guidelines recommended for reporting animal research

Laboratory animals	M. musculus, Nude Mouse NU/NU (Charles River), 8-12 weeks of age, M/F equally distributed across treatment groups
Wild animals	No wild animals were used in this study.
Field-collected samples	No samples collected in fields were used in this study.
Ethics oversight	Institutional Animal Care and Use Committee at the CU-AMC (IACUC protocol 00432)

Note that full information on the approval of the study protocol must also be provided in the manuscript.

ChIP-seq

Data deposition

- [x] Confirm that both raw and final processed data have been deposited in a public database such as GEO.
- [ ] Confirm that you have deposited or provided access to graph files (e.g. BED files) for the called peaks.

Data access links<br>*May remain private before publication.*	https://www.ncbi.nlm.nih.gov/geo/query/acc.cgi?&acc=GSE191150<br>Peaks were not called because of low enrichment scores. Therefore, no BED files are provided.
Files in database submission	p53_ChiPseq.K1.DMSO.395.bs1.deeptools.normalized.bigwig<br>p53_ChiPseq.K1.DMSO.399.bs1.deeptools.normalized.bigwig<br>p53_ChiPseq.K1.GSK.397.bs1.deeptools.normalized.bigwig<br>p53_ChiPseq.K1.GSK.401.bs1.deeptools.normalized.bigwig<br>p53_ChiPseq.K1.input.K1.bs1.deeptools.normalized.bigwig<br>p53_ChiPseq.K1.Nutlin.396.bs1.deeptools.normalized.bigwig<br>p53_ChiPseq.K1.Nutlin.400.bs1.deeptools.normalized.bigwig<br>p53_ChiPseq.K1.NutlinGSK.398.bs1.deeptools.normalized.bigwig<br>p53_ChiPseq.K1.NutlinGSK.402.bs1.deeptools.normalized.bigwig

p53_ChiPseq.TPC1.DMSO.387.bs1.deeptools.normalized.bigwig
p53_ChiPseq.TPC1.DMSO.391.bs1.deeptools.normalized.bigwig
p53_ChiPseq.TPC1.GSK.389.bs1.deeptools.normalized.bigwig
p53_ChiPseq.TPC1.GSK.393.bs1.deeptools.normalized.bigwig
p53_ChiPseq.TPC1.input.TPC1.bs1.deeptools.normalized.bigwig
p53_ChiPseq.TPC1.Nutlin.388.bs1.deeptools.normalized.bigwig
p53_ChiPseq.TPC1.Nutlin.392.bs1.deeptools.normalized.bigwig
p53_ChiPseq.TPC1.NutlinGSK.390.bs1.deeptools.normalized.bigwig
p53_ChiPseq.TPC1.NutlinGSK.394.bs1.deeptools.normalized.bigwig
387_S1_L001_R1_001.fastq.gz
388_S2_L001_R1_001.fastq.gz
389_S3_L001_R1_001.fastq.gz
390_S4_L001_R1_001.fastq.gz
391_S5_L001_R1_001.fastq.gz
392_S6_L001_R1_001.fastq.gz
393_S7_L001_R1_001.fastq.gz
394_S8_L001_R1_001.fastq.gz
395_S9_L001_R1_001.fastq.gz
396_S10_L001_R1_001.fastq.gz
397_S11_L001_R1_001.fastq.gz
398_S12_L001_R1_001.fastq.gz
399_S13_L001_R1_001.fastq.gz
400_S14_L001_R1_001.fastq.gz
401_S15_L001_R1_001.fastq.gz
402_S16_L001_R1_001.fastq.gz
K1_S17_L001_R1_001.fastq.gz
R11960_S1_L003_R1_001.fastq.gz
R11961_S2_L003_R1_001.fastq.gz
R11962_S3_L003_R1_001.fastq.gz
R11963_S4_L003_R1_001.fastq.gz
R11964_S5_L003_R1_001.fastq.gz
R11965_S6_L003_R1_001.fastq.gz
R11966_S7_L003_R1_001.fastq.gz
R11967_S8_L003_R1_001.fastq.gz
R12062_S9_L004_R1_001.fastq.gz
R12063_S10_L004_R1_001.fastq.gz
R12064_S11_L004_R1_001.fastq.gz
R12065_S12_L004_R1_001.fastq.gz
R12066_S13_L004_R1_001.fastq.gz
R12066_S29_L005_R1_001.fastq.gz
R12067_S14_L004_R1_001.fastq.gz
R12067_S30_L005_R1_001.fastq.gz
R12068_S15_L004_R1_001.fastq.gz
R12068_S31_L005_R1_001.fastq.gz
R12069_S16_L004_R1_001.fastq.gz
R12069_S32_L005_R1_001.fastq.gz
TPC1_S18_L001_R1_001.fastq.gz
TPC1_K1_HTSeq_counts.txt

Genome browser session (e.g. UCSC): https://genome.ucsc.edu/s/andrysik/p53_TPC1_K1

Methodology

Replicates: Replicates (marked rep1, rep2) were generated in independent experiments.

Sequencing depth:
Sample:,Cell line - treatment,total reads,mapped reads
387,TPC1 - DMSO - rep1,1.72E+07,1.42E+07
388,TPC1 - Nutlin - rep1,1.49E+07,1.22E+07
389,TPC1 - GSK - rep1,1.82E+07,1.04E+07
390,TPC1 - NutlinGSK - rep1,1.86E+07,1.61E+07
391,TPC1 - DMSO - rep2,2.12E+07,1.84E+07
392,TPC1 - Nutlin - rep2,2.28E+07,1.98E+07
393,TPC1 - GSK - rep2,2.09E+07,1.83E+07
394,TPC1 - NutlinGSK - rep2,1.95E+07,1.73E+07
395,K1 - DMSO - rep1,1.76E+07,1.50E+07
396,K1 - Nutlin - rep1,1.92E+07,1.64E+07
397,K1 - GSK - rep1,1.99E+07,1.68E+07

398,K1 - NutlinGSK - rep1,1.97E+07,1.68E+07
399,K1 - DMSO - rep2,2.70E+07,2.31E+07
400,K1 - Nutlin - rep2,1.85E+07,1.58E+07
401,K1 - GSK - rep2,2.88E+07,2.46E+07
402,K1 - NutlinGSK - rep2,2.10E+07,1.80E+07
K1input,,1.97E+07,1.68E+07
TPC1input,,2.13E+07,1.84E+07

Antibodies	p53 (DO-1), Calbiochem, OP43
Peak calling parameters	Peaks were not called because of low enrichment scores.
Data quality	Due to low enrichment score of the ChIP-seq data, no peak calling was performed and data use was limited to qualitative analysis of p53 occupancy at ATF3 and ATF4 loci.
Software	FASTQC (v0.11.5), FastQ Screen (v0.11.0), FASTQ-MCF from EAUtils (v1.05), Bowtie2 (v2.2.9), Samtools (v1.5), Picard (v2.9.4), deepTools105 (version 2.2.2)

Flow Cytometry

Plots

Confirm that:

- [x] The axis labels state the marker and fluorochrome used (e.g. CD4-FITC).
- [x] The axis scales are clearly visible. Include numbers along axes only for bottom left plot of group (a 'group' is an analysis of identical markers).
- [x] All plots are contour plots with outliers or pseudocolor plots.
- [x] A numerical value for number of cells or percentage (with statistics) is provided.

Methodology

Sample preparation	The fraction of apoptotic cells was determined by Annexin V-FITC/PI assay. Briefly, cells harvested by trypsinization were resuspended in Annexin-V binding buffer (10 mM HEPES pH 7.4, 140 mM NaCl, 2.5 mM CaCl2). Approximately 2x105 cells were labeled with Annexin-V-FITC (Invitrogen) and PI (10 ug/ml, Millipore-Sigma) for 15 minutes in the dark before flow cytometric analysis (Accuri C6, Becton Dickinson).<br>To analyze mitochondrial membrane potential cells were trypsinized and resuspended in cultivation media. An aliquot of approximately 5x105 cells per sample was mixed with Tetramethylrhodamine, Ethyl Ester, Perchlorate (TMRE, Thermo Fisher, 100 nM final concentration) solution, incubated for 10 minutes in the dark, and analyzed by flow cytometer. Reactive oxygen species levels were measured using 6-chloromethyl-2',7'-dichlorodihydrofluorescein diacetate, acetyl ester (CM-H2DCFDA). Briefly, trypsinized cells were resuspended in the cultivation media, combined with CM-H2DCFDA solution (10 uM final concentration), and incubated for 15 minutes in the dark. At least 104 particles per sample were analyzed for fluorescence intensity in the FL1 channel (533/30 nm). Proteasomal activity was analyzed with Me4BodipyFL-Ahx3Leu3VS fluorescent probe (abbreviated as Me4BodipyFL). After the treatment period, cultivation media in both TPC1 and HCT116 cells was replaced with pre-warmed 0.5 uM of Me4BodipyFL in PBS for 1 hour. Next, cells were harvested by trypsinization, and fluorescence was measured by flow cytometry. Intracellular levels of Fe2+ ions were measured with FerroOrange probe (Dojindo). Briefly, trypsinized cells were resuspended in HBSS buffer, pelleted, resuspended in serum-free DMEM media, and stained with 1 uM FerroOrange dye for 15 min at 37C.
Instrument	Becton Dickinson Accuri C6
Software	Accuri c6 (version 1.0.264.21), FlowJo (visualization, version 10.4.2)
Cell population abundance	Tissue culture samples only.
Gating strategy	Live/apoptotic cells were gated by FCC/SCC signal, quadrants to distinguish viable, early and late apoptotic cells were used for Annexin V-FITC/propidium iodide staining. An example of gating strategy is provided in Supplementary Information file.

- [x] Tick this box to confirm that a figure exemplifying the gating strategy is provided in the Supplementary Information.
