# Supplementary material for: PPM1D suppresses p53-dependent transactivation and cell death by inhibiting the Integrated Stress Response
Source: Nat Commun. 2022 Dec 1;13:7400. doi: 10.1038/s41467-022-35089-5 (PMC9715646; doi:10.1038/s41467-022-35089-5)
Supplement: Supplementary file 8 — Source Data [file 41467_2022_35089_MOESM8_ESM.zip › Source Data/Source Data C - raw GTEx analysis data.zip!/Andrysik - Source data - raw GTEx analysis data/EIF2B1/EIF2B1.normalVStumor.expression.boxplot.pdf]

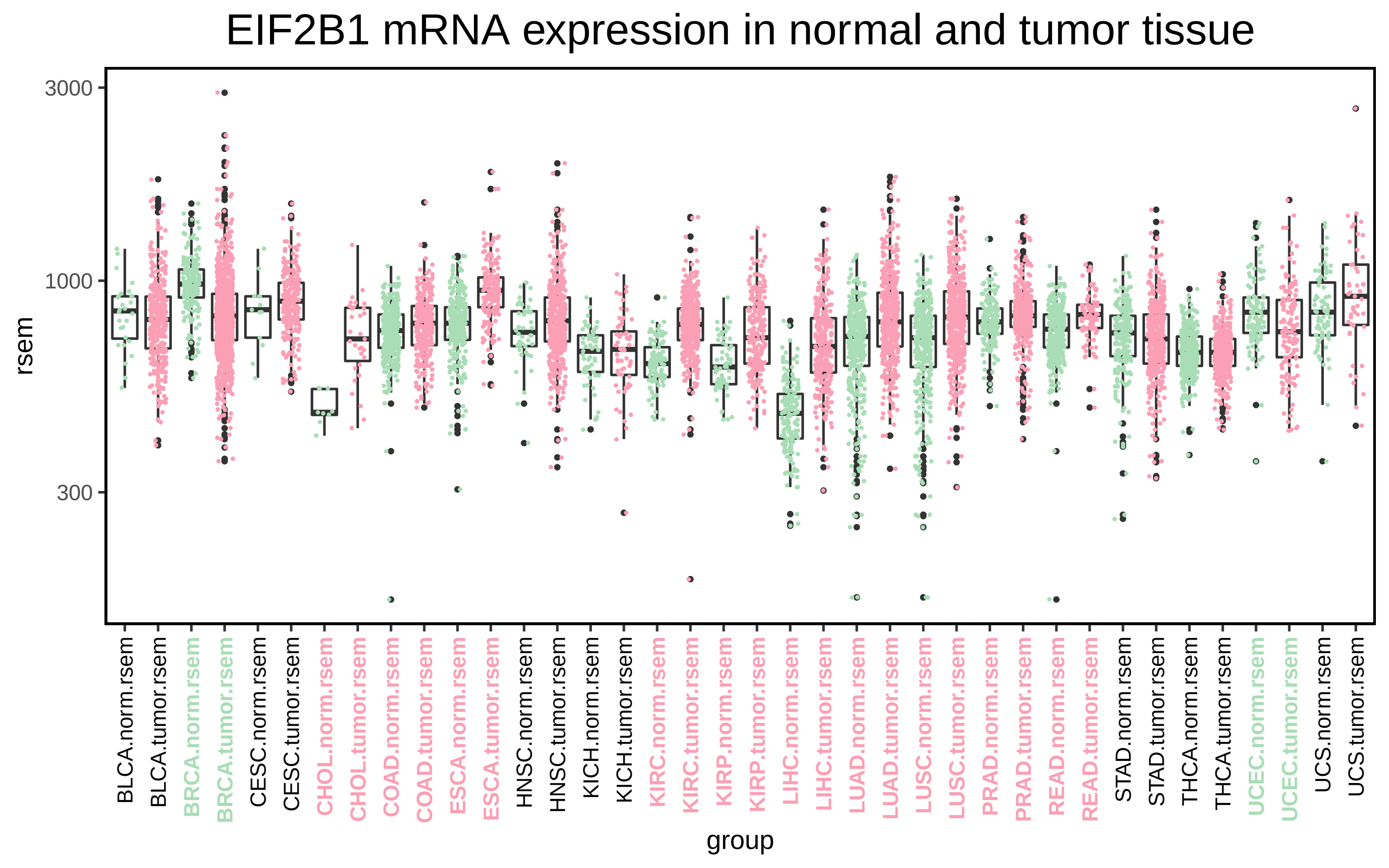
EIF2B1 mRNA expression in normal and tumor tissue
3000
1000
300
rsem
group
BLCA.norm.rsem
BLCA.tumor.rsem
BRCA.norm.rsem
BRCA.tumor.rsem
CESC.norm.rsem
CESC.tumor.rsem
CHOL.norm.rsem
CHOL.tumor.rsem
COAD.norm.rsem
COAD.tumor.rsem
ESCA.norm.rsem
ESCA.tumor.rsem
HNSC.norm.rsem
HNSC.tumor.rsem
KICH.norm.rsem
KICH.tumor.rsem
KIRC.norm.rsem
KIRC.tumor.rsem
KIRP.norm.rsem
KIRP.tumor.rsem
LIHC.norm.rsem
LIHC.tumor.rsem
LUAD.norm.rsem
LUAD.tumor.rsem
LUSC.norm.rsem
LUSC.tumor.rsem
PRAD.norm.rsem
PRAD.tumor.rsem
READ.norm.rsem
READ.tumor.rsem
STAD.norm.rsem
STAD.tumor.rsem
THCA.norm.rsem
THCA.tumor.rsem
UCEC.norm.rsem
UCEC.tumor.rsem
UCS.norm.rsem
UCS.tumor.rsem
