# Supplementary material for: PPM1D suppresses p53-dependent transactivation and cell death by inhibiting the Integrated Stress Response
Source: Nat Commun. 2022 Dec 1;13:7400. doi: 10.1038/s41467-022-35089-5 (PMC9715646; doi:10.1038/s41467-022-35089-5)

EIF2S2 mRNA expression in normal and tumor tissue

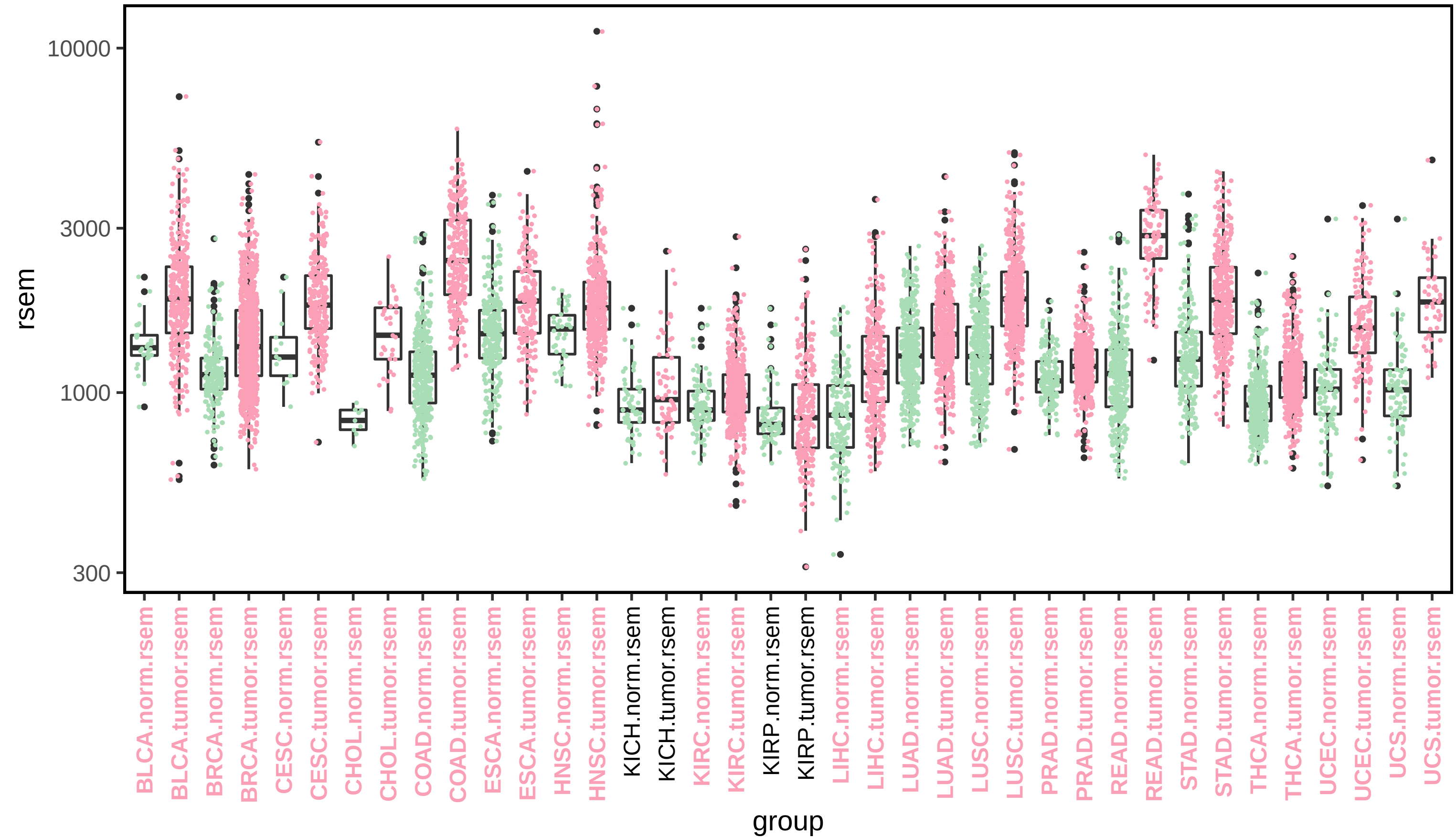

Supplement: Supplementary file 8 — Source Data [file 41467_2022_35089_MOESM8_ESM.zip › Source Data/Source Data C - raw GTEx analysis data.zip!/Andrysik - Source data - raw GTEx analysis data/EIF2S2/EIF2S2.normalVStumor.expression.boxplot.pdf]
